# Mechanical forces remodel the cardiac extracellular matrix during zebrafish development

**DOI:** 10.1242/dev.202310

**Published:** 2024-07-10

**Authors:** Alessandra Gentile, Marga Albu, Yanli Xu, Newsha Mortazavi, Agatha Ribeiro da Silva, Didier Y. R. Stainier, Felix Gunawan

**Affiliations:** ^1^ Department of Developmental Genetics, Max Planck Institute for Heart and Lung Research, Bad Nauheim 61231, Germany; ^2^ Institute of Cell Biology, Faculty of Medicine, University of Münster, Münster 48149, Germany

**Keywords:** Biomechanical forces, Cardiac ECM remodeling, Cardiac development, Zebrafish cardiogenesis

## Abstract

The cardiac extracellular matrix (cECM) is fundamental for organ morphogenesis and maturation, during which time it undergoes remodeling, yet little is known about whether mechanical forces generated by the heartbeat regulate this remodeling process. Using zebrafish as a model and focusing on stages when cardiac valves and trabeculae form, we found that altering cardiac contraction impairs cECM remodeling. Longitudinal volumetric quantifications in wild-type animals revealed region-specific dynamics: cECM volume decreases in the atrium but not in the ventricle or atrioventricular canal. Reducing cardiac contraction resulted in opposite effects on the ventricular and atrial ECM, whereas increasing the heart rate affected the ventricular ECM but had no effect on the atrial ECM, together indicating that mechanical forces regulate the cECM in a chamber-specific manner. Among the ECM remodelers highly expressed during cardiac morphogenesis, we found one that was upregulated in non-contractile hearts, namely *tissue inhibitor of matrix metalloproteinase 2* (*timp2*). Loss- and gain-of-function analyses of *timp2* revealed its crucial role in cECM remodeling. Altogether, our results indicate that mechanical forces control cECM remodeling in part through *timp2* downregulation.

## Teaser

**Summary:** Mechanical forces regulate zebrafish cardiac extracellular matrix volume dynamics in a region-specific manner, an effect mediated at least in part by Tissue inhibitor of metalloproteinase 2b (Timp2b).

## INTRODUCTION

The extracellular matrix (ECM) forms an integral component of all tissues. It provides an anchoring substrate to which cells adhere and influences the distribution of signaling molecules that modulate cell behaviors (Walma and Yamada, 2020; Derrick and Noel, 2021). Tight regulation of ECM components gives rise to distinct ECM, and consequently tissue, properties: for example, production and cross-linking of fibrillar collagens increase tensile strength in connective tissue ECM, whereas high amounts of proteoglycans and glycosaminoglycans confer a soft ECM in the brain (Handorf et al., 2015). In the vertebrate heart, a layer of specialized ECM called the cardiac jelly or cardiac ECM (cECM) separates the first two cell layers: the outer-lining cardiomyocytes (CMs) and inner-lining endocardial cells (EdCs) (Derrick and Noel, 2021; Gunawan et al., 2021). The cECM promotes essential processes during development, including the migration of CMs and EdCs to form the cardiac tube (Trinh and Stainier, 2004; Totong et al., 2011) and of a subset of EdCs to form the cardiac valves (Walsh and Stainier, 2001; Smith et al., 2011; De Angelis et al., 2017; Gunawan et al., 2019; Hernandez et al., 2019). It also mediates communication between CMs and EdCs (Bornhorst et al., 2019) and the incorporation of second heart field cells into the atrium (Derrick et al., 2021). As further evidence of the importance of the cECM, variants of several ECM genes in humans are associated with congenital heart defects (Liu et al., 1997; Pöschl et al., 2004; Lincoln et al., 2006; Zhou et al., 2024).

Structural proteins (e.g. collagens, laminins and elastin) and associated signaling molecules (e.g. TGFβ, Wnt and Notch) regulate the biophysical properties of various epithelial and endothelial tissues as well as short- and long-range signaling between cells. To regulate their amount and activation state, these proteins are post-translationally cleaved by metalloproteinases of the MMP (matrix metalloproteinase) and ADAMTS (a disintegrin and metalloproteinase with thrombospondin motifs) families (Lu et al., 2011). The function of MMP and ADAMTS proteins can be inhibited by tissue inhibitors of metalloproteinases (TIMPs), which bind and block their catalytic and proteolytic domains. In mouse, mutations in single TIMP genes lead to mild, non-lethal defects that include dilated cardiomyopathy in global *Timp3* mutants (Fedak et al., 2004). Combined mutations in all four mouse TIMP genes (*Timp1-4*) lead to decreased life expectancy and postnatal bone growth defects (Saw et al., 2019), suggesting that TIMPs can functionally compensate for each other during mouse development. Notably, in pathological conditions, single TIMP mutants exhibit significant impairment in their recovery after injury or infection. In mouse, *Timp2* mutations have been associated with poor response to myocardial infarction, an effect that was alleviated by MMP inhibition (Kandalam et al., 2011). *Timp3* and *Timp4* deficiencies in mouse also exacerbate maladaptive remodeling post myocardial injury, leading to excessive fibrosis (Fan et al., 2014; Takawale et al., 2014; Yarbrough et al., 2014). However, increased levels of *Timp1* are also associated with myocardial fibrosis (Takawale et al., 2017), suggesting that the complex functions of TIMPs render the tight regulation of their expression to be crucial. Importantly, the cellular processes that TIMPs modulate in the heart and how TIMPs regulate the cECM during development remain largely unknown.

The ECM is known to influence the mechanical properties of tissues, but is itself also regulated by mechanical stress. The influence of mechanical forces on ECM dynamics has been well described in cell culture; exposure of various cell types to cyclic mechanical stress or fluid flow leads to increased expression, or cross-linking, of fibrillar matrix components, MMPs or TIMPs (Chiquet-Ehrismann et al., 1994; Kubow et al., 2015; Saw et al., 2019). The link between mechanical forces and the cECM remains largely unexplored in the developing heart. This gap is important to address as biomechanical forces brought on by the rhythmic cardiac contraction and blood flow play important roles in cardiac tissue patterning and growth (Bartman et al., 2004; Collins and Stainier, 2016; Haack and Abdelilah-Seyfried, 2016; Rasouli and Stainier, 2017; Gunawan et al., 2021). Biomechanical forces in the heart are known to induce the recruitment and/or activation of membrane receptors and ion channels (Just et al., 2011; Duchemin et al., 2019; Juan et al., 2023, 2024) that signal to cytosolic and nuclear factors and affect cell fates and behaviors, as well as tissue integrity (Gentile et al., 2021). Biomechanical forces also pattern the cardiac transcriptional landscape by regulating mechanosensitive transcription factors, such as Klf2, Egr3 and Wwtr1, that promote the formation of the cardiac valves (Steed et al., 2016; Ribeiro da Silva et al., 2024) and trabecular network (Lai et al., 2018), and influence calcium wave generation in the EdCs and CMs (Fukui et al., 2021). However, whether and how mechanical forces influence cECM volume and remodeling during development remains poorly understood.

Here, we use the zebrafish as a model to study the cECM because it offers two important advantages. First, the zebrafish is the only vertebrate model in which the cECM can be imaged and tracked in 3D in living animals, which enables continuous cECM volume quantification (Derrick and Noël, 2021; Sánchez-Posada and Noël, 2024 preprint). Second, blocking (or increasing) cardiac contraction up to 120 hours post fertilization (hpf) does not lead to death or gross morphological abnormalities, allowing one to assess the role of biomechanical forces on cECM dynamics. Focusing on cardiac development between 48 and 96 hpf, when cardiac valves and trabeculae take shape, our 3D quantitative analyses of the cECM show that its volume remains constant in the ventricle and atrioventricular canal (AVC), indicating that the ventricular and AVC cECM undergo flattening or spreading as the heart grows. Only the atrial cECM volume decreases during this developmental period, suggesting that the atrial cECM is lost over time. We also found that decreased cardiac contraction or increased heart rate led to abnormal remodeling of the cECM in a chamber-specific manner. Furthermore, our data show that cECM remodeling depends at least in part on *timp2b*, expression of which is repressed by cardiac contraction.

## RESULTS

### The cardiac ECM undergoes region-specific remodeling

**Fig. 1.**
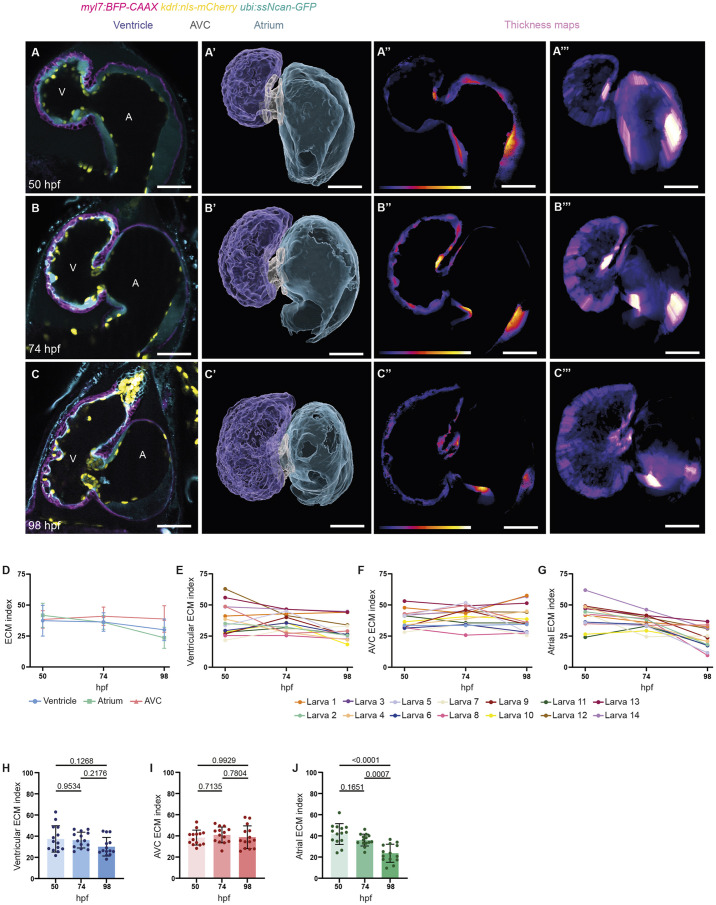
**cECM remodeling during cardiac morphogenesis.** (A-C‴) cECM dynamics in the same animal from 50 to 98 hpf. Single-plane images (A-C) and 3D surface renderings (A′-C′) of confocal images, as well as single-plane (A″-C″) and maximum intensity projection (A‴-C‴) thickness maps of wild-type hearts at 50, 74 and 98 hpf. Color brightness in the thickness maps (A″-C‴) correlates with tissue/ECM thickness. White indicates higher tissue/ECM thickness, and purple indicates lower tissue/ECM thickness. *Tg(myl7*:BFP-CAAX*)* expression marks the myocardial chambers, *Tg(kdrl:*nls-mCherry*)* expression marks the endocardial chambers, and *Tg(ubb:*ssNcan-GFP*)* expression labels hyaluronic acid, which is present in the cardiac ECM. (D-J) Quantification of the cECM index (percentage of the ECM volume relative to the total cardiac tissue volume) in the ventricle (D,E,H), the AVC (D,F,I) and the atrium (D,G,J) from 50 to 98 hpf. Between 50 and 98 hpf, the cECM index remains constant in the ventricle and AVC and progressively decreases in the atrium. *n*=14 (D-J); plot values represent mean±s.d. (D,H-J); *P*-values determined by one-way ANOVA followed by multiple comparisons with Dunn test (H-J). Scale bars: 30 µm. A, atrium; AVC, atrioventricular canal; V, ventricle.

Thinning of the cECM, particularly in the ventricle prior to trabeculation, has previously been documented (Rasouli and Stainier, 2017; del Monte-Nieto et al., 2018), but cECM volume dynamics at later stages has only recently begun to be investigated (Sánchez-Posada and Noël, 2024 preprint). We focused on developmental stages when cardiac valve formation, trabeculation and chamber ballooning take place, and performed a longitudinal and region-specific quantitative analysis of cECM volume. Using a transgenic line that expresses the hyaluronic acid (HA)-binding domain of Neurocan under a ubiquitous promoter, *Tg(ubi:ssNcan-GFP)* (Grassini et al., 2018), combined with myocardial *Tg(myl7:BFP-CAAX)* and endocardial *Tg(kdrl:nls-mCherry)* transgenic reporters, we imaged the same animals every 24 h from 50 to 98 hpf. *Tg(ubb:*ssNcan-GFP*)* expression was present throughout almost all extracellular space, including in very narrow spaces between the CMs and EdCs (Fig. 1A-C‴), and so it was used to label the cECM. cECM thickness progressively decreased in all three cardiac regions examined, the atrium, AVC, and ventricle (Fig. 1A″-C‴). We then performed volumetric measurements of the cECM in these three regions (Fig. S1A-C). We also determined the total cardiac tissue volume bounded by the myocardium (Fig. S1D-F) and the cardiac lumen volume (Fig. S1G-I) to record cardiac growth over time. To correlate changes in cECM volume with cardiac chamber growth, we calculated the ratio of the cECM volume relative to the total cardiac tissue volume, referred to as the ECM index. Surprisingly, only in the atrium did the total cECM volume and index become significantly progressively smaller (Fig. 1D,G,J; Fig. S1C). The ventricular ECM volume and index did not significantly change at the time points observed, although the ventricular ECM index exhibited a decreasing trend between 74 and 98 hpf (Fig. 1D,E,H; Fig. S1A). The AVC cECM volume and index remained mostly unchanged (Fig. 1D,F,I; Fig. S1B). These results suggest that even though the distance between the CMs and EdCs became narrower over developmental time, the ventricular and AVC cECM volumes did not strongly decrease but instead became mainly redistributed over a larger surface area.

### Mechanical forces regulate cardiac ECM remodeling

**Fig. 2.**
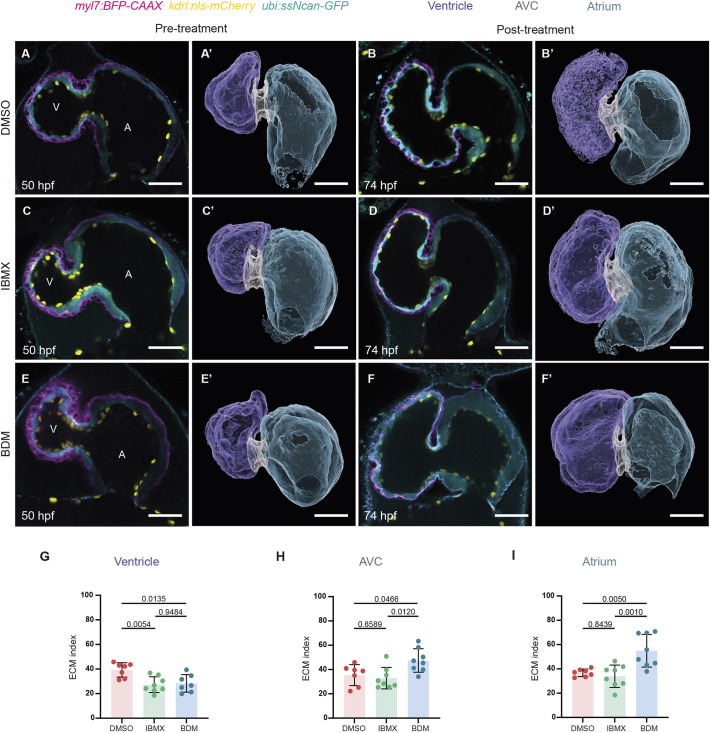
**Mechanical forces regulate cECM dynamics in a region-specific manner.** (A-F′) Wild-type hearts before (50 hpf; A,A′,C,C′,E,E′) and after (74 hpf; B,B′,D,D′,F,F′) 24-h treatments with DMSO control (A-B′), IBMX (C-D′) or BDM (E-F′). Single-plane images are shown in A-F and 3D surface renderings in A′-F′. Images before and after treatment were taken of the same animals. (G-I) cECM index of IBMX- or BDM-treated hearts compared with DMSO-treated hearts at 74 hpf. A reduction of the ventricular ECM index was observed in IBMX- and BDM-treated hearts (G). For the AVC (H) and atrial (I) ECM indices, increases were observed in BDM-treated hearts, whereas no changes were observed in IBMX-treated hearts (H,I). *n*=7 for DMSO, 8 for IBMX, and 7 for BDM (G-I); plot values represent mean±s.d.; *P*-values determined by one-way ANOVA followed by multiple comparisons with Dunn test. Scale bars: 30 µm. A, atrium; AVC, atrioventricular canal; V, ventricle.

We then investigated the role of cardiac contraction and blood flow in regulating the volume of the cECM. We incubated 50 hpf zebrafish embryos with compounds known to reduce cardiac contraction [2,3-butanedione monoxime (BDM)] or increase heart rate [3-isobutyl-1-methylxanthine (IBMX)] (De Luca et al., 2014; Fukuda et al., 2019) for 24 h. We imaged the same hearts pre- and post-treatment and determined the cECM index. Quantification of the chamber (Table 1; Fig. S2A-C; Table S1) and lumen (Table 1; Fig. S2D-F; Table S1) volumes showed that the hearts did not collapse following BDM or IBMX treatment; instead, the ventricular chamber and lumen dilated following treatment (Table 1; Fig. S2A,D; Table S1). The total cECM volumes were not altered upon IBMX treatment, but significantly increased in all chambers upon BDM treatment (Table 1; Fig. S2G-I; Table S1). However, these changes in ECM volumes might be a secondary effect due to the chamber dilations when contraction was reduced; hence, we quantified the cECM index as a parameter to correlate ECM volumes with changes in chamber volumes. Interestingly, compared with control (Fig. 2A-B′,G), the ventricular cECM index became significantly reduced in both decreased cardiac contraction and increased heart rate conditions (Fig. 2C-F′,G; Table 1; Table S1), indicating that the ventricular ECM is particularly susceptible to changes in biomechanical forces. The increase of total cECM volume in the ventricle (Fig. S2G) did not match the scale of cardiac chamber dilation (Fig. S2A) upon loss of contraction. In contrast to the ventricular cECM index, the atrial and AVC cECM index significantly increased when contraction was blocked (Fig. 2E-F′,H,I; Table 1; Table S1) but remained unchanged upon increased heart rate (Fig. 2C-D′,H,I; Table 1; Table S1). Altogether, these results indicate that biomechanical forces affect the remodeling or distribution of the cECM in a region-specific manner.

**Table 1.**
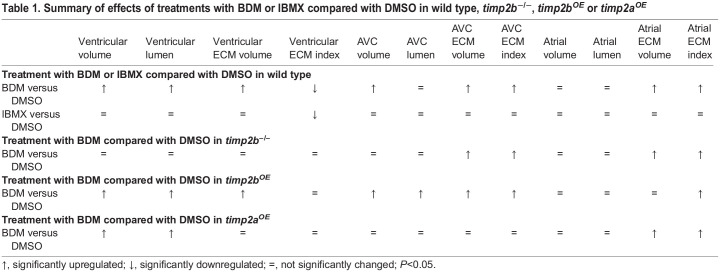
Summary of effects of treatments with BDM or IBMX compared with DMSO in wild type, *timp2b*^−/−^, *timp2b*^*OE*^ or *timp2a*^*OE*^

### Expression of the ECM remodeling gene *timp2b* is regulated by cardiac forces

**Fig. 3.**
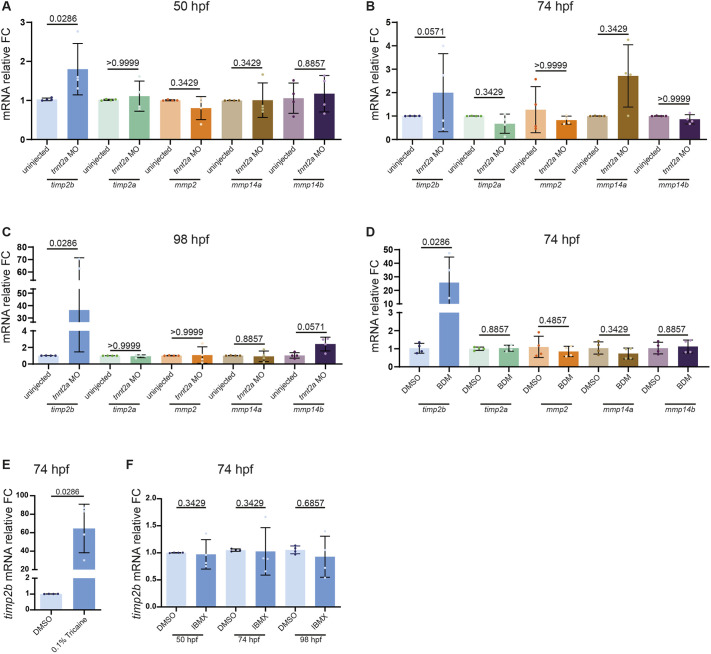
***timp2b* expression is upregulated in hearts contracting at a reduced rate.** (A-C) Relative mRNA levels of *timp2b*, *timp2a*, *mmp2*, *mmp14a* and *mmp14b* from extracted hearts of *tnnt2a* morphants and uninjected controls at 50 (A), 74 (B) and 98 hpf (C). (D) Relative mRNA levels of *timp2b*, *timp2a*, *mmp2*, *mmp14a* and *mmp14b* from extracted hearts of 74 hpf zebrafish treated for 24 h with BDM or DMSO. (E) Relative *timp2b* mRNA levels from extracted hearts of 74 hpf zebrafish treated for 24 h with Tricaine or DMSO. (F) Relative *timp2b* mRNA levels from extracted hearts of 50, 74 and 98 hpf zebrafish treated for 24 h with IBMX or DMSO. *timp2b* expression is significantly upregulated upon reduced cardiac contraction (*tnnt2a* morpholino, BDM and Tricaine treatments), but remains unaffected upon increased heart rate (IBMX treatment). *n*=4 biological replicates, 25 hearts each. Plot values represent mean±s.d.; *P*-values determined by Mann–Whitney *U* test. FC, fold change. See Table S6 for Ct values.

To investigate the mechanisms by which biomechanical forces regulate the cECM, we focused on factors involved in ECM remodeling, particularly members of the MMP and TIMP families. We first identified the most highly expressed MMP and TIMP genes (*mmp2*, *mmp14a*, *mmp14b*, *timp2a* and *timp2b*) in the developing zebrafish heart from our previous transcriptomic dataset (Gunawan et al., 2019). We then performed qPCR analysis to assess the expression levels of these genes in wild-type and non-contractile, i.e. *tnnt2a* morphant, hearts. Surprisingly, we found that *timp2b* expression was higher in non-contractile hearts compared with control at 50, 74 and 98 hpf (Fig. 3A-C; Table S6). Our qPCR data showed that among the selected MMP and TIMP genes, *timp2b* was the only gene that was consistently upregulated at all time points analyzed (Fig. 3A-C; Table S6).

As the *tnnt2a* morpholino completely blocks the onset of cardiac contraction, which might produce secondary effects on *timp2b* expression, we tested whether reducing cardiac contraction in a temporally restricted window also increased *timp2b* expression. We incubated the animals from 50 to 74 hpf, the time point we focused on for our imaging analysis (Fig. 2), in BDM or Tricaine, two chemicals that inhibit cardiac contraction through different molecular mechanisms. We consistently observed increased *timp2b* expression at 74 hpf in both BDM and Tricaine treatments (Fig. 3D,E; Table S6), an increase that was even greater than that observed at 50 and 74 hpf in *tnnt2a* morphants (Fig. 3A,B). These results confirm that *timp2b* expression is abnormally upregulated upon loss of cardiac contraction. The greater increase of *timp2b* expression in BDM-treated animals than in *tnnt2a* morphants might be due to a partial dependence of the initiation of cardiac *timp2b* transcription on cardiac contraction. We further tested whether increased heart rate led to reduced *timp2b* expression, but found no significant change in *timp2b* expression upon IBMX treatment (Fig. 3F).

### Mechanical force-dependent cardiac ECM remodeling partly relies on Timp2b activity

We analyzed the expression pattern of *timp2b* in the developing heart using fluorescence *in situ* hybridization and found *timp2b* expression in both EdCs and CMs at 48 and 72 hpf (Fig. S3A-B″), although with a noticeably stronger expression in EdCs than in CMs at 72 hpf (Fig. S3B-B″). Although our results show that *timp2b* expression is negatively regulated by cardiac biomechanical forces, it has also been reported in mouse that loss of *Timp2* expression is detrimental for cardiac physiology (Kandalam et al., 2010, 2011; Fan et al., 2014). Thus, we wanted to assess the effects of losing *timp2b* function in zebrafish and generated a mutant allele using CRISPR/Cas9 technology. This allele, *timp2b^bns617^*, contains a 5-bp insertion in exon 2 and is predicted to encode a Timp2 protein that contains only about half of its MMP-binding domain (Fig. S3C,D).

**Fig. 4.**
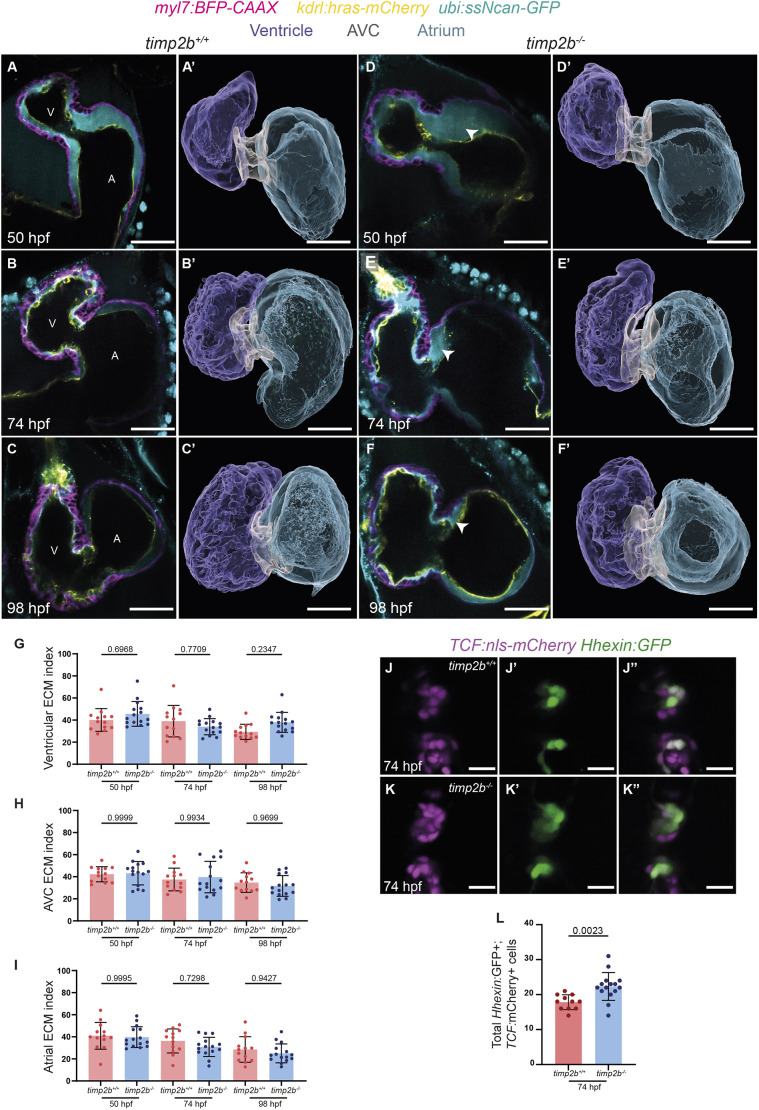
***timp2b* mutant hearts exhibit increased cECM thickness in the atrial side of the AVC.** (A-F′) Single-plane images and 3D surface renderings of *timp2b^+/+^* (A-C′) and *timp2b^−/−^* (D-F′) sibling hearts at 50, 74 and 98 hpf. cECM thickness is increased in the atrial side of the AVC (white arrowheads in D-F) in *timp2b^−/−^* hearts compared with *timp2b^+/+^* sibling hearts. (G-I) Quantification of the ventricular, AVC and atrial ECM indices at 50, 74 and 98 hpf. (J-K″) Single-plane images of AV valves from 74 hpf *timp2b^−/−^* and *timp2b^+/+^* sibling larvae. Valve EdCs are labeled by *Tg(TCF:*nls-mCherry*)* and *Tg(Hhexin:*GFP*)* expression. (L) Quantification of the total number of *Tg(Hhexin*:GFP)^+^; *Tg(TCF:*nls-mCherry*)*^+^ cells at 74 hpf shows an increased number of valve cells in *timp2b^−/−^* compared with *timp2b^+/+^* sibling larvae. *n*=13 for *timp2b^+/+^* and 15 for *timp2b*^−/−^ (G-I); *n*=11 for *timp2b^+/+^* and 15 for *timp2b^−/−^* (L). Plot values represent mean±s.d.; *P*-values determined by one-way ANOVA followed by multiple comparisons with Dunn test (G-I) and by unpaired two-tailed Student's *t*-test (L). Scale bars: 30 µm (A-F′); 20 µm (J-K″). A, atrium; AVC, atrioventricular canal; V, ventricle.

*timp2b* homozygous mutants do not present gross morphological defects up to 120 hpf, including unaffected body length and size. However, by 98 hpf, about 50% of *timp2b* mutants exhibited pericardial edema, which typically indicates cardiovascular defects (Fig. S3D, red asterisk). Closer inspection of cardiac morphology at 50, 74 and 98 hpf show that neither the cardiac tissue as bounded by the myocardium (Fig. S3E-G) nor the lumen volume (Fig. S3E″-G″) in any of the three cardiac regions presented significant differences between *timp2b* mutants and their homozygous wild-type siblings. We also examined the cECM index, volume and thickness in *timp2b* mutants and their homozygous wild-type siblings. Interestingly, *timp2b* mutants consistently display increased cECM thickness on the atrial side of the AVC compared with their wild-type siblings (Fig. 4A-F′; arrowheads point to the expanded cECM in Fig. 4D-F). This increased thickness was observed at 50, 74 and 98 hpf (Fig. 4D-F). However, the ventricular (Fig. 4G; Fig. S3E′), AVC (Fig. 4H; Fig. S3F′) and atrial (Fig. 4I; Fig. S3G′) cECM indices and volumes were not significantly changed in *timp2b* mutants compared with their wild-type siblings. Altogether, these data suggest that loss of *timp2b* affects the distribution but not the volume of cECM, which becomes abnormally concentrated in the atrial side of the AVC.

As the effects of the *timp2b* mutation were primarily observed in the AVC ECM, we also examined cardiac valve formation in these mutants. The AVC EdCs that form the AV valve upregulate markers that clearly distinguish them from other endocardial cells (Steed et al., 2016; Gunawan et al., 2020). In zebrafish, the AVC EdCs that form the valve upregulate *Tg(Hhexin:*GFP*)* expression (Gunawan et al., 2020) as well as *Tg(TCF:*nls-mCherry*)* expression, the latter of which labels cells with active Wnt/β-catenin signaling (Moro et al., 2012). We crossed these transgenic reporters to *timp2b* mutants and observed at 74 hpf a significantly higher number of valve EdCs (*Hhex:*GFP^+^; *TCF:*nls-mCherry^+^ cells) in *timp2b* mutants compared with their homozygous wild-type siblings (Fig. 4J-L). These results show that loss of *timp2b* causes an expansion of the AVC ECM as well as an expansion of valve EdCs, or mis-patterning of the endocardial tissue in the AVC.

**Fig. 5.**
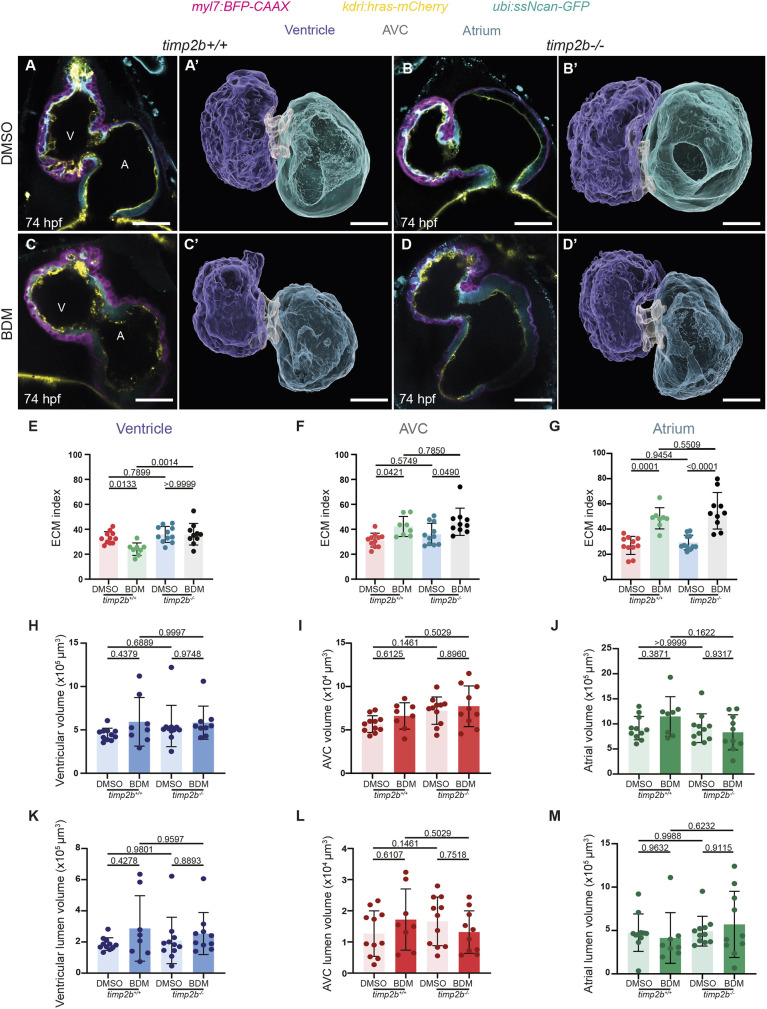
**Mechanical forces regulate cECM organization at least in part through *timp2b.*** (A-D′) 74 hpf *timp2b^+/+^* and *timp2b^−/−^* sibling hearts following treatment with DMSO and BDM starting at 50 hpf. Single-plane images and 3D surface renderings of 74 hpf *timp2b^+/+^* (A,A′,C,C′) and *timp2b^−/−^* (B,B′,D,D′) sibling hearts following treatment with DMSO (A-B′) or BDM (C-D′) starting at 50 hpf. (E-G) Quantification of ventricular (E), AVC (F) and atrial (G) ECM indices of 74 hpf *timp2b^+/+^* and *timp2b^−/−^* sibling hearts following treatment with BDM compared with DMSO controls starting at 50 hpf. BDM-induced reduction of the cECM index in *timp2b^+/+^* ventricles is not apparent in *timp2b^−/−^* ventricles (E), indicating that *timp2b* expression is needed for the mechanical force-induced loss of the ventricular cECM index. cECM index in the AVC (F) and atrium (G) is still significantly higher when BDM treatment was used instead of DMSO treatment on *timp2b^+/+^* and *timp2b^−/−^* siblings. (H-J) Quantification of the ventricular (H), AVC (I) and atrial (J) volumes. (K-M) Quantification of the ventricular (K), AVC (L) and atrial (M) lumen volumes. *n*=11 for DMSO, *timp2b^+/+^*; 8 for BDM, *timp2b^+/+^*; 11 for DMSO, *timp2b^−/−^*; and 10 for BDM, *timp2b^−/−^*. Plot values represent mean±s.d.; *P*-values determined by one-way ANOVA followed by multiple comparisons with Dunn test. Scale bars: 30 µm. A, atrium; AVC, atrioventricular canal; V, ventricle.

As *timp2b* expression was upregulated when cardiac contraction was lost (Fig. 3), we hypothesized that when *timp2b* function was lost, slowing down cardiac contraction might not lead to significant changes in cECM volumes. We tested this hypothesis by treating *timp2b* mutants with DMSO as control or with BDM starting at 50 hpf and quantifying the cECM index (Fig. 5A-G; Tables 1, 2; Table S2) and cECM volume (Tables 1, 2; Fig. S3H-J; Table S2) at 74 hpf, along with the total cardiac volume (Fig. 5H-J; Tables 1, 2; Table S2) and lumen volume (Fig. 5K-M; Tables 1, 2; Table S2). Interestingly, loss of *timp2b* rescued the cECM index reduction in the ventricle of BDM-treated larvae. Whereas BDM-treated *timp2b^+/+^* siblings had a smaller ventricular cECM index, BDM-treated *timp2b^−/−^* larvae had a cECM index comparable to that of DMSO-treated *timp2b^+/+^* and DMSO-treated *timp2b^−/−^* larvae (Fig. 5E; Tables 1, 2; Table S2). This effect was not observed for the atrial or AVC ECM, as both *timp2b^+/+^* and *timp2b^−/−^* larvae displayed significantly increased AVC and atrial ECM indices when treated with BDM compared with DMSO (Fig. 5F,G; Tables 1, 2; Table S2). These results suggest that the inhibition of *timp2b* by contraction-mediated mechanical forces is required to maintain the proper amount of cECM in the ventricle. However, cardiac contraction may exert its effects on AVC and atrial cECM through additional mechanisms that might be at least partly independent of *timp2b* function.

**Table 2.**
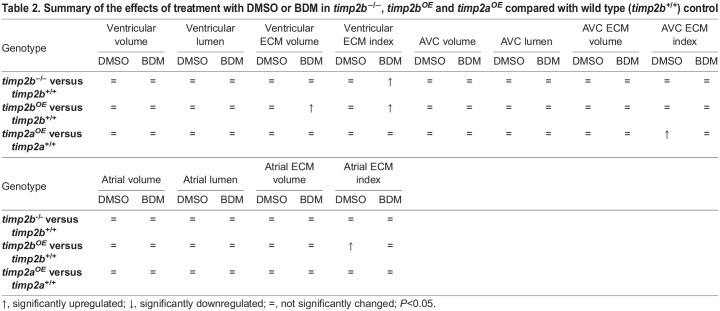
Summary of the effects of treatment with DMSO or BDM in *timp2b*^−/−^, *timp2b*^*OE*^ and *timp2a*^*OE*^ compared with wild type (*timp2b*^+/+^) control

### *timp2* overexpression leads to enlarged cardiac valves and expanded AVC and atrial ECM

**Fig. 6.**
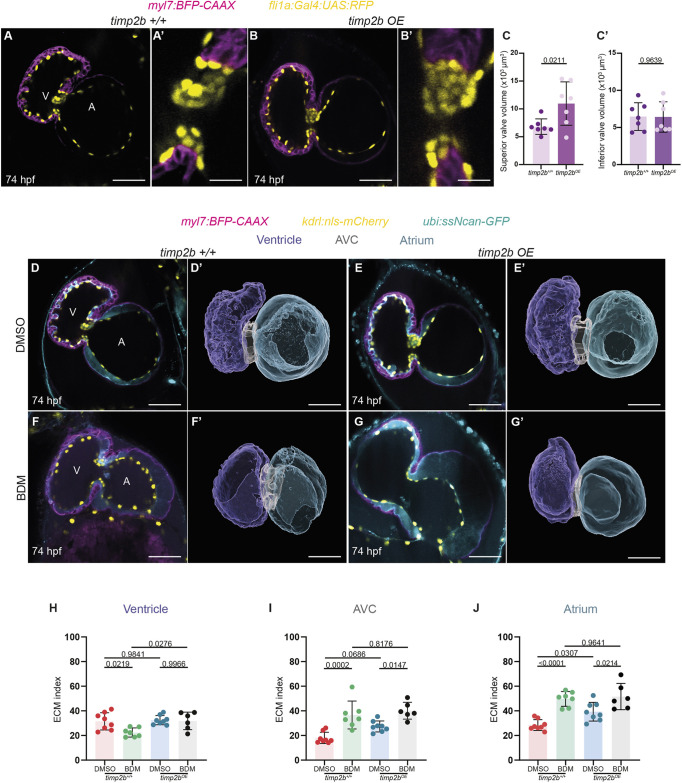
***timp2* overexpression leads to enlarged cardiac valves and increased AVC and atrial ECM index.** (A-B′) Single-plane images and close-up of AV valves of 74 hpf control (A,A′) and *timp2b-*overexpressing [*Tg(fli1a:Gal4); Tg(UAS:timp2b-p2a-GFP)*; B,B′] hearts. There is noticeable enlargement of the valve observed in *timp2b*-overexpressing hearts (B′) compared with control (A′). (C,C′) Quantification of 3D valve tissue volumes in control and *timp2b*-overexpressing larvae at 74 hpf. When *timp2b* is overexpressed in EdCs, a significantly increased valve tissue volume is observed in the superior leaflet, but not in the inferior leaflet. (D-G′) Single-plane images and 3D surface renderings of 74 hpf control (D,D′,F,F′) and *timp2b-*overexpressing (E,E′,G,G′) hearts treated with DMSO (D-E′) or BDM (F-G′) starting at 50 hpf. Upon BDM treatment, *timp2b* overexpression leads to severe cardiac defects (G,G′) that appear worse than those in BDM-treated control larvae (F,F′). (H-J) Quantification of cECM index of 74 hpf control and *timp2b-*overexpressing hearts treated with DMSO or BDM starting at 50 hpf. Increased AVC (I) and atrial (J) cECM index is observed in *timp2b-*overexpressing hearts treated with DMSO. *n*=7 for *timp2b^+/+^* and 7 for *timp2b^OE^* (C,C′); 8 for DMSO, *timp2b^+/+^*; 7 for BDM, *timp2b^+/+^*; 8 for DMSO, *timp2b^OE^*; and 6 for BDM, *timp2b^OE^*. Plot values represent mean±s.d.; *P*-values determined by unpaired two tailed Student's *t*-test (C,C′) or one-way ANOVA followed by multiple comparisons with Dunn test (H-J). Scale bars: 20 µm (A,B); 10 µm (A′,B′); 30 µm (D-G′). A, atrium; AVC, atrioventricular canal; V, ventricle.

Blocking cardiac contraction led to increased *timp2b* expression as well as cECM disorganization, which could be partly rescued in *timp2b* mutants. We therefore investigated the effects of overexpressing *timp2a/b* in the heart on cECM organization and tissue morphology. As the most noticeable effects of *timp2b* disruption involved endocardial-derived cardiac valve formation, we hypothesized that Timp2 primarily functions in the endocardium. The zebrafish Timp2a and Timp2b proteins share a high degree of similarity, with 65% identity and 78% similarity in the amino acids composing their protease inhibitory domains (Fig. S4A). We overexpressed either *timp2a* or *timp2b* specifically in EdCs [*Tg(fli1:Gal4); Tg(UAS:timp2a-p2a-GFP)* or *Tg(UAS:timp2b-p2a-GFP)*]. Interestingly, the cardiac valves in *timp2a*- and *timp2b-*overexpressing larvae appeared abnormally large compared with control (Fig. 6A-B′; Fig. S5A-B′). Volumetric quantifications confirmed a significant increase of the superior valve, but not inferior valve, leaflets when either *timp2a* or *timp2b* was overexpressed (Fig. 6C,C′; Fig. S5C,C′). These results suggest that as both loss and gain of *timp2b* lead to enlarged valves, tightly regulated expression of *timp2* is required to constrain valve tissue and valve ECM volume.

We further analyzed the effects of increased *timp2a* or *timp2b* on the cECM volume and index, as well as total cardiac volume and lumen volume, in normally contractile and low-contractile conditions (Fig. 6D-J; Figs S4B-J, S5D-S; Tables 1, 2; Tables S3, S4). Even in control DMSO conditions, the AVC and atrial ECM indices consistently increased when either *timp2a* or *timp2b* was overexpressed compared with control (Fig. 6I,J; Fig. S5L,P; Tables 1, 2; Tables S3, S4), confirming that high expression of *timp2a/b* mostly affects these regions. The ventricular ECM index was not significantly changed when *timp2a* or *timp2b* was overexpressed (Fig. 6H; Fig. S5H; Tables 1, 2; Tables S3, S4). Strikingly, *timp2a* or *timp2b* overexpression combined with BDM treatment led to severely enhanced cardiac defects, with absence of normal structures, including cardiac valves and trabeculae (Fig. 6G,G′; Fig. S5G,G′). These results suggest that combined contraction slowdown and high *timp2a/b* expression are detrimental to cardiac development. Compared with DMSO, BDM treatment led to significantly increased AVC and atrial ECM indices that were comparable between wild-type and *timp2a-* or *timp2b-*overexpressing larvae (Fig. 6I,J; Fig. S5L,P; Tables 1, 2; Tables S3, S4). Although increased *timp2a/b* expression appeared to alleviate the ventricular ECM index reduction observed in wild type upon BDM treatment (Fig. 6H; Fig. S5H; Tables 1, 2; Tables S3, S4), we postulate that, unlike *timp2b* mutant rescue of BDM-treated hearts, the severe defects observed in BDM-treated *timp2a-* or *timp2b*-overexpressing hearts led to a secondary rescue of the ECM volumes. Our results show that cardiac ECM organization is highly dependent on proper regulation of *timp2a/b* expression.

## DISCUSSION

Here, we identified a crucial role for mechanical forces in ECM remodeling during cardiac morphogenesis. Although several studies have alluded to the two-dimensional spatial reduction of the ECM between EdCs and CMs during cardiac development (Rasouli and Stainier, 2017; del Monte-Nieto et al., 2018; Grassini et al., 2018; Qi et al., 2022), only one recent study (Sánchez-Posada and Noël, 2024 preprint) has tested in which cardiac regions or how the three-dimensional cECM reorganization occurs. Using transgenic lines to track CMs, EdCs, and the HA-binding peptide in the cECM, we identified region-specific reduction of the cECM starting at 50 hpf. Interestingly, although we and others have observed a thinning of the gap between EdCs and CMs, our results show that neither the absolute cECM volume, nor the cECM volume relative to the chamber volume (ECM index) changes significantly in the ventricle or AVC; these results suggest that the cECM is not lost, but instead undergoes redistribution over a growing surface area in the developing heart. cECM significantly decreased only in the atrium, which may be driven by degradation, redistribution out of the heart, or a lack of ECM factor synthesis or stabilization.

Furthermore, we found that increasing the heart rate or reducing cardiac contraction affects the cECM volume in a chamber-specific manner. During the time when the ventricle undergoes substantial morphogenetic changes to form trabeculae, increasing the heart rate or reducing cardiac contraction led to decreased ventricular cECM volume, highlighting the importance of the correct amount of mechanical forces during ventricular morphogenesis. Decreasing cardiac contraction led to increased AVC and atrial cECM, but elevated heart rate did not affect it, suggesting a degree of robustness in these regions in maintaining their ECM volume. These differences in cECM regulation by cardiac forces might be due to the complex trabecular formation occurring in the ventricle, whereby some CMs undergo depolarization, delamination and proliferation (Jiménez-Amilburu et al., 2016; Rasouli and Stainier, 2017; Uribe et al., 2018; Priya et al., 2020). These dynamic cellular processes mediate ‘inside-out’ effects via integrins on ECM organization and degradation (Lu et al., 2011), potentially rendering the ventricular cECM more susceptible to perturbations in cardiac contraction.

While we primarily focused on the effects of contraction-induced biomechanical forces in cECM regulation, dysregulation of cECM reorganization is known to impact cardiac morphogenesis (Derrick et al., 2021, 2022), thereby causing a feedback loop that leads to heart malformations. Indeed, local non-uniform ECM remodeling has been implicated in various morphogenetic events that globally affect organ formation, including the folding of epithelial sheets during intestinal (Ramadan et al., 2022), brain (Long et al., 2018; Güven et al., 2020) and ear canal (Munjal et al., 2021) development, apical constriction during neurulation, and branching during salivary gland formation (Wang et al., 2021). The cardiac region-specific differences in cECM volume might be not only correlated with local morphogenetic events, including ventricular trabeculation and valve formation, but also provide asymmetric extracellular tension to modulate the looping and ballooning processes that sculpt the heart.

Our study uncovered Timp2b as an important link between the cECM, biomechanical forces, and cardiac morphogenesis. We found that *timp2b* is in fact one of the few ECM factors for which expression is inhibited by mechanical forces. These results open interesting questions regarding how contractility affects not only *timp2b* expression but also the molecular properties that regulate ECM volume. As cECM protein composition evolves over time from a soft, glycoprotein-based ECM to a stiff, fibrillar collagen-based ECM (Hinton et al., 2006; Lockhart et al., 2011; Hulin et al., 2019; Gunawan et al., 2020), it will be interesting to address the links between the composition, changing physical volumes, and biomechanical properties of the cECM that promote cardiac morphogenesis.

In conclusion, our work provides one of the first characterizations of cECM dynamics in 3D and underlines the need for a comprehensive analysis of ECM volume to improve our understanding of ECM synthesis, degradation, and spatial distribution. The use of other approaches, for instance quantifying the negative, unlabeled space between EdCs and CMs (Sánchez-Posada and Noël, 2024 preprint), would complement our study with the aim of elucidating ECM volume dynamics in the heart and various other tissues, and might mitigate potential caveats associated with transgenic lines used to fluorescently mark the ECM. Future investigations of cECM volume dynamics during other temporal windows or specific morphogenetic processes, such as valvulogenesis and trabeculation, will further elucidate how cell–ECM interactions promote cardiac development.

## MATERIALS AND METHODS

### Zebrafish husbandry

Zebrafish husbandry was performed in accordance with institutional (Max-Planck-Gesellschaft) and national (German) ethical and animal welfare regulation. Larvae were raised under standard conditions. Adult zebrafish were maintained in 3.5 l tanks at a stock density of ten zebrafish/l with the following parameters: water temperature 27-27.5°C; light:dark cycle 14:10; pH 7.0-7.5; conductivity 750–800 µS/cm. Zebrafish were fed three to five times a day, depending on age, with granular and live food (*Artemia salina*). Health monitoring was performed at least once a year. All embryos used in this study were raised at 28°C and staged at 75% epiboly for synchronization. All procedures performed on animals conform to the guidelines from Directive 2010/63/EU of the European Parliament on the protection of animals used for scientific purposes and were approved by the Animal Protection Committee (Tierschutzkommission) of the Regierungspräsidium Darmstadt (reference: B2/1218).

### Zebrafish lines

The following transgenic and mutant lines were used in this study: *Tg(myl7:BFP-CAAX)^bns193^* (Guerra et al., 2018); *Tg(kdrl:nls-mCherry)^is4Tg^* (Wang et al., 2010); *Tg(kdrl:hras-mCherry)^s896Tg^* (Chi et al., 2008); *Tg(ubi:ssNcan-GFP)^uq25bhTg^* (Grassini et al., 2018); *Tg(fli1a:Gal4)^ubs4^* (Zygmunt et al., 2011); *Tg(Hhexin:GFP)*^bns321^ (Gunawan et al., 2020); *Tg(7xTCF-Xla.Sia:NLS-mCherry)^ia5^* (Moro et al., 2012), abbreviated as *Tg*(*TCF:nls-mCherry)*; *Tg(UAS:timp2a-p2a-GFP)^bns443^* (this study); *Tg(UAS:timp2b-p2a-GFP)^bns675^* (this study); and *timp2b^bns617^* (this study).

### Generation of *timp2a* and *timp2b* overexpression transgenic lines

To generate the *timp2a* overexpression line, the full coding sequence was amplified by PCR using the following primers: forward 5′-ATGAAGAGCGTCAGGAGCTGT-3′ and reverse 5′-AGGGTCTTCCACATCCA-3′. The 660 bp amplicon was cloned into pT2-UAS plasmid upstream of a P2A linker and GFP. To generate the *timp2b* overexpression line, the full coding sequence was amplified by PCR using the following primers: forward 5′-GTCGACCACCATGAGTATGTC-3′ and reverse 5′-TGGTTCCTCGATGTCCATGAAC-3′. The 651 bp amplicon was cloned into pT2-UAS plasmid upstream of a P2A linker and GFP. All cloning experiments were performed using ColdFusion Cloning (System Biosciences). The plasmid was then injected into *Tg(fli1a:Gal4)* embryos at the one-cell stage (25 pg/embryo) together with *Tol2* mRNA (25 pg/embryo) to generate *Tg(UAS:timp2a-p2a-GFP)* and *Tg(UAS:timp2b-p2a-GFP)*.

### Generation of *timp2b^bns617^*

*timp2b* mutants were generated using CRISPR/Cas9 technology. The guide RNA (gRNA) sequence was designed using the CRISPOR program (http://crispor.tefor.net/) to target exon 1 of the *timp2b* gene (5′-GTCACCGGCAATGACGCTTA-3′), leading to a 5 bp insertion. The gRNAs were transcribed using a MegaShortScript T7 Transcription Kit (Thermo Fisher Scientific). *cas9* mRNA was transcribed using a MegaScript T3 Transcription Kit (Thermo Fisher Scientific) using pT3TS-nCas9n as a template. Both gRNA and Cas9 RNAs were purified with an RNA Clean and Concentrator Kit (Zymo Research). gRNAs (∼12.5 pg/embryo) and *cas9* mRNA (∼300 pg/embryo) were co-injected at the one-cell stage. High resolution melt analysis was used to determine the efficiency of sgRNA. Primers are listed in Table S5. All imaging and analyses of *timp2b* mutants were carried out in the F3 generation or later.

The resulting mutant allele is a 5 bp insertion (lower case letters) and a 1 bp substitution (C>T in bold) into exon 2 of the *timp2b* gene. Wild-type exon 2: TCATCAGAGCAAAAGTCGTCGGAAGAAAGGAGGTGGTCACCGGCAATGACGCTTATGGCTATCCAATCAAAATGATCCGATACGATGTCAAACAGTTGAAG. *timp2b^bns617^* exon 2: TCATCAGAGCAAAAGTCGTCGGAAGAAAGGAGGTGGTCACCGGCAATGACGgg**T**TggaTATGGCTATCCAATCAAAATGATCCGATACGATGTCAAACAGTTGAAG.

### Chemical treatments and morpholino injections

Heart contraction was decreased or increased by chemical treatments from 48 to 72 hpf. First, 48 hpf PTU-treated wild-type embryos were imaged and then recovered in single Petri dishes with 10 ml of egg water before starting the chemical treatments. To decrease the heart rate, embryos were treated with 15 mM of BDM (Sigma-Aldrich, B0753) or 0.1% w/v of Tricaine (Pharmaq); to increase the heart rate, embryos were treated with 100 µM of IBMX (Sigma-Aldrich, I5879). BDM was dissolved in water, and IBMX and Tricaine in DMSO. DMSO was used as a vehicle control at a final concentration of 0.1%. Treated and control embryos were incubated in single Petri dishes at 28°C until the next day and then imaged with a confocal microscope. For *timp2b* mutants (Figs 4, 5), or *timp2-*overexpressing (Fig. 6; Fig. S5) larvae, the animals were not treated with Tricaine or *tnnt2a* morpholino, and imaged only after BDM treatment.

*tnnt2a* morpholino (5′-CATGTTTGCTCTGATCTGACACGC-3′) was injected into one-cell-stage embryos at 0.3 ng per embryo, and used to slow down contraction from the onset of cardiac development (Sehnert et al., 2002).

### Imaging

Confocal microscopes were used to image stopped hearts. Embryos were mounted in 1% low-melting agarose with 0.2% Tricaine, and the stopped hearts were imaged using a Zeiss LSM880 confocal microscope with a 20× dipping lens using the Fast Airyscan mode. To follow the same animal over a period of 24 or 48 h, embryos and larvae were recovered in single Petri dishes and imaged the next day. For whole-mount *in situ* hybridization, embryos were mounted on 1% agar and imaged using a Nikon SMZ25 microscope with a 40× objective.

To image beating hearts, a spinning disk microscope was used to acquire videos from 50 to 98 hpf at 100 frames per second with a 40× water immersion lens (Zeiss Cell Observer spinning disk microscope).

### Quantitative PCR analysis

Dissected hearts or whole 50 hpf embryos were homogenized in TRIzol using a NextAdvance Bullet Blender homogenizer, followed by standard phenol/chloroform extraction. At least 500 ng total RNA was used for reverse transcription using a Maxima First Strand cDNA synthesis kit (Thermo Fisher Scientific). For all experiments, DyNAmo ColorFlash SYBR Green qPCR Mix (Thermo Fisher Scientific) was used on a CFX connect real-time System (Bio-Rad) with the following program: pre-amplification 95°C for 7 min, amplification 95°C for 10 min and 60°C for 30 min (repeated 39 times), melting curve 60°C to 92°C with increment of 1.0°C each 5 min. Each point in the dot plots represents a biological replicate. Gene expression values were normalized using the housekeeping gene *rpl13a* and fold changes were calculated using the 2^−ΔΔ*C*t^ method. Primers are listed in Table S5. Ct values are listed in Table S6.

### Image analysis

Imaris software was used to obtain the chamber and cardiac ECM volumes. The volume of the ventricle, AVC and atrium was manually segmented in 3D using the cardiomyocyte membrane marker (*myl7:*BFP-CAAX expression) as a template. This chamber segmentation obtained from the membrane marker was used to mask the cECM *Tg(ubi:*ssNcan-GFP*)* expression from the same heart in 3D, in each of the chambers, and obtain the cECM volume. To obtain the cECM heatmap, the Biofilm analysis Imaris XTension was used (https://imaris.oxinst.com/open/view/biofilm-analysis).

### Randomization procedures

All experiments using *timp2b* mutants were randomized as follows: animals from heterozygous crosses were collected, imaged and analyzed, and subsequently genotyped. Transgenic animals were selected by fluorescence before imaging, and therefore could not be randomized. The investigators were unaware of treatment group allocation during experiments and outcome assessment whenever possible.

### Fluorescence *in situ* hybridization

The following primers were used to generate the DNA template for *in situ* RNA probes to detect *timp2b* expression: forward 5′-ATGAGTATGTCTCGGTCAGTTCC-3′, reverse 5′-TAATACGACTCACTATAGGGGGATGTATTCTCATGGTTCCTCGATGTCCA-3′.

Fluorescence *in situ* hybridization was performed on 48 hpf embryos and 72 hpf larvae, as previously described (Gunawan et al., 2019). MF20 primary antibody (mouse monoclonal; 14-6503-82, eBioscience; 1:500) was used to label CMs.

### Statistical analysis

Every sample group was tested for Gaussian distribution using the D'Agostino-Pearson omnibus normality test. For all the experiments that passed the normality test, all samples were further analyzed using parametric tests: *P*-values were determined using unpaired, two-tailed Student's *t*-test for comparison of two samples or the one-way ANOVA test followed by correction for multiple comparisons with Dunn's Test for comparison of three samples. For all the experiments that did not pass the normality test, all samples were further analyzed using non-parametric tests: *P*-values were determined using the Mann–Whitney test for comparison of two samples. A significant difference was considered when the *P*-value was less than 0.05.

## Supplementary Material

10.1242/develop.202310_sup1 Supplementary information
