# Supplementary material for: Mechanical forces remodel the cardiac extracellular matrix during zebrafish development
Source: Development. 2024 Jul 10;151(13):dev202310. doi: 10.1242/dev.202310 (PMC11266798; doi:10.1242/dev.202310)
Supplement: Supplementary information [file develop-151-202310-s1.pdf]

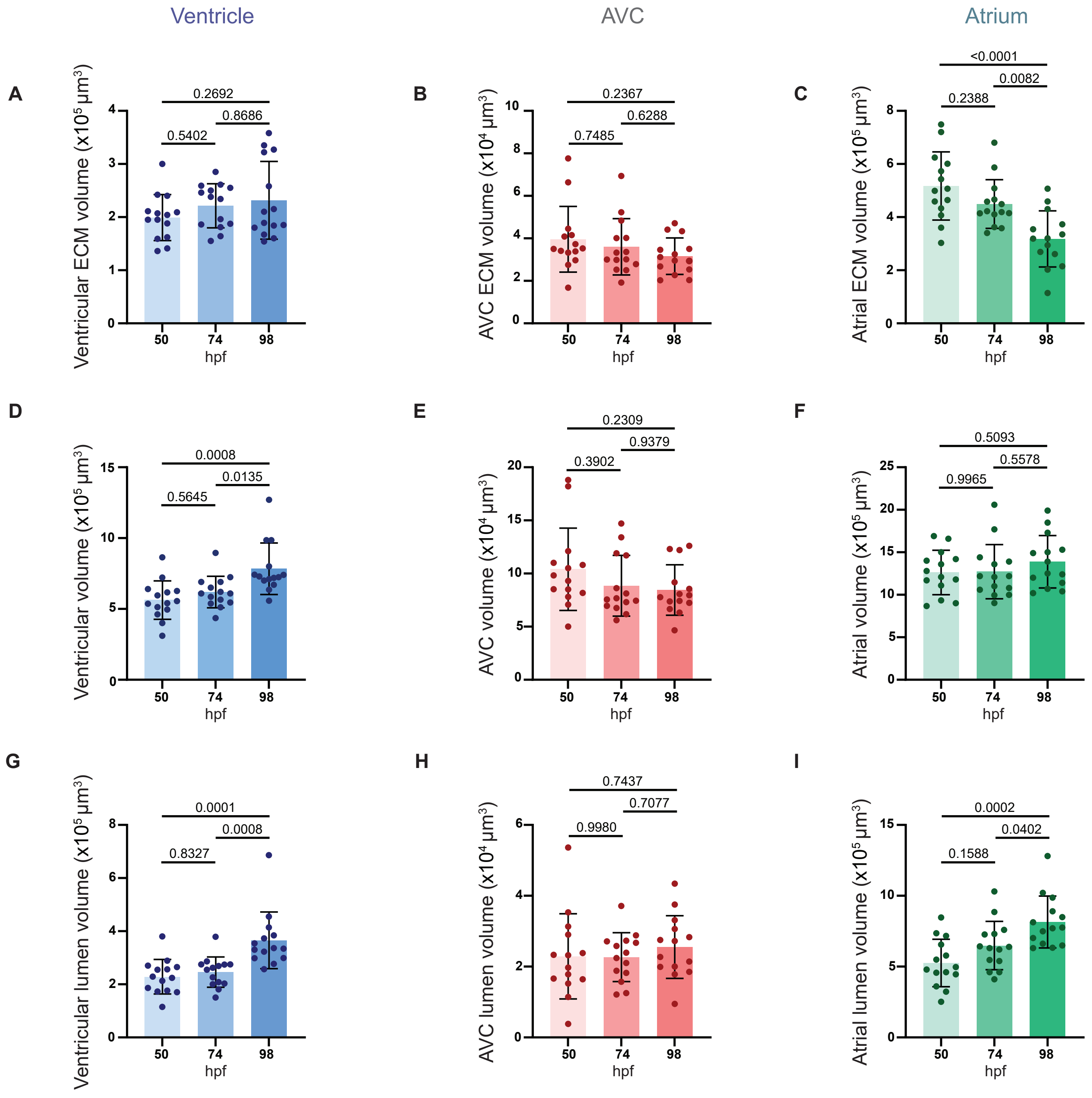

Fig. S1. Total ECM, regional and lumen volume dynamics during wild-type development.

A-C) Quantification of the total ventricular, AVC and atrial ECM volumes. Over time, total cECM volume remains constant in the ventricle (A) and the AVC (B), and decreases in the atrium (C). **D-F)** Quantification of the total ventricular (D), AVC (E) and atrial (F) volumes, as outlined by *Tg(myl7:*BFP-CAAX) expression. Chamber volume growth is most prominent in the ventricle (D). **G-I)** Quantification of the ventricular (G), AVC (H) and atrial (I) lumen volumes. The ventricular and atrial lumen volumes progressively expand. n=14 (A-I); plot values represent means ± S.D.; p-values determined by one-way ANOVA followed by multiple comparisons with Dunn test.

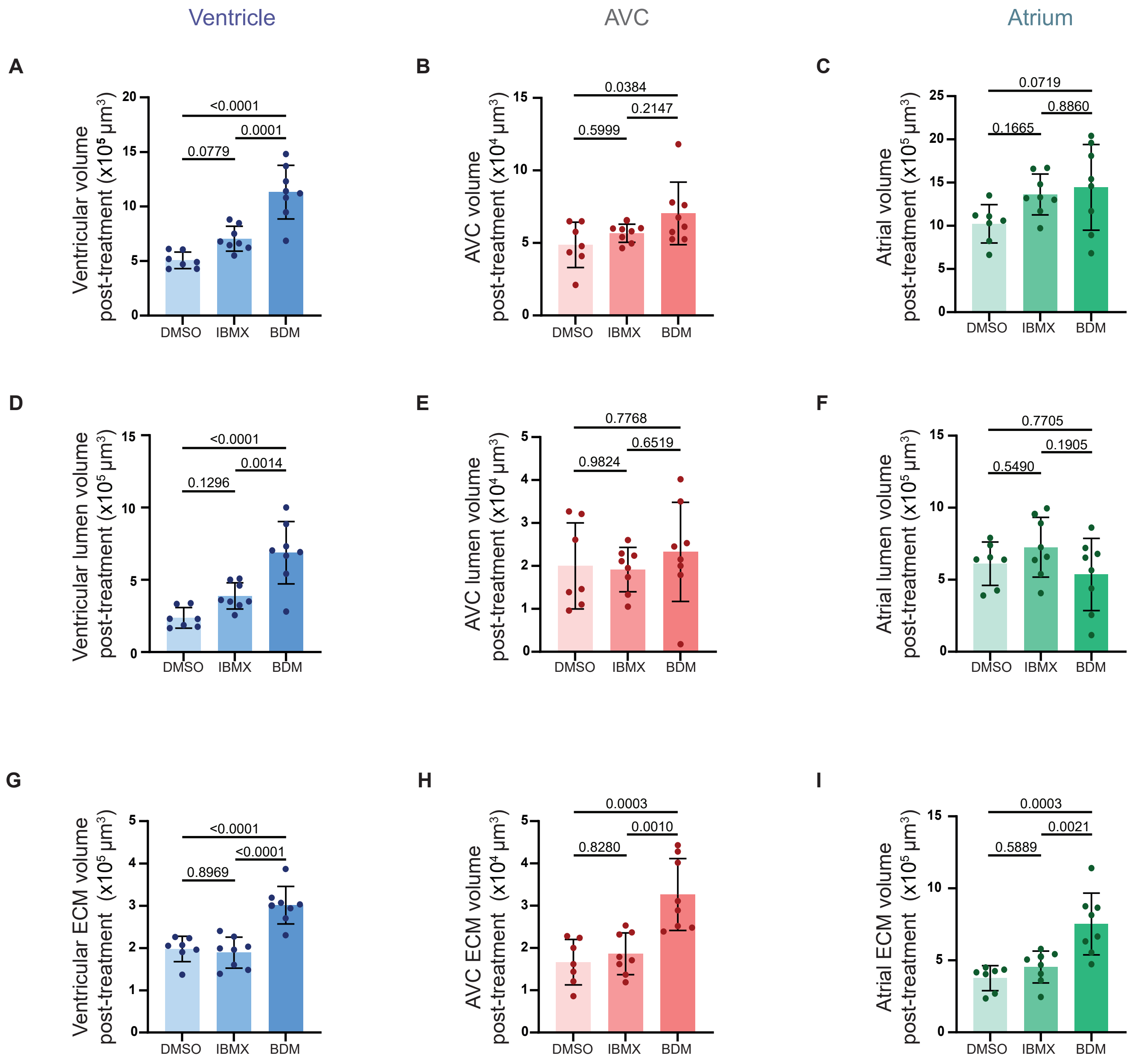

Fig. S2. Total ECM, regional and lumen volume dynamics in response to altered cardiac contraction.

A-C) Quantification of ventricular (A), AVC (B), and atrial (C) volumes in 74 hpf larvae after 24 hours of BDM or IBMX treatment compared with DMSO. The ventricle and AVC volumes are significantly higher in BDM-treated larvae compared with control. No other regional volumes are affected by BDM or IBMX treatment. **D-F)** Quantification of ventricular (D), AVC (E), and atrial (F) lumen volumes in 74 hpf larvae after 24 hours of BDM or IBMX treatment compared with DMSO. Ventricular lumen expansion is observed upon BDM treatment. **G-I)** Quantification of total ECM volume in 74 hpf ventricle (G), AVC (H), and atrium (I) after 24 hours of BDM or IBMX treatment compared with DMSO. Increased total ECM volume is observed in all chambers after BDM treatment, but not IBMX treatment. n=7 for DMSO, 8 for IBMX, and 7 for BDM (G-I); plot values represent means ± S.D.; p-values determined by one-way ANOVA followed by multiple comparisons with Dunn test.

Development • Supplementary information

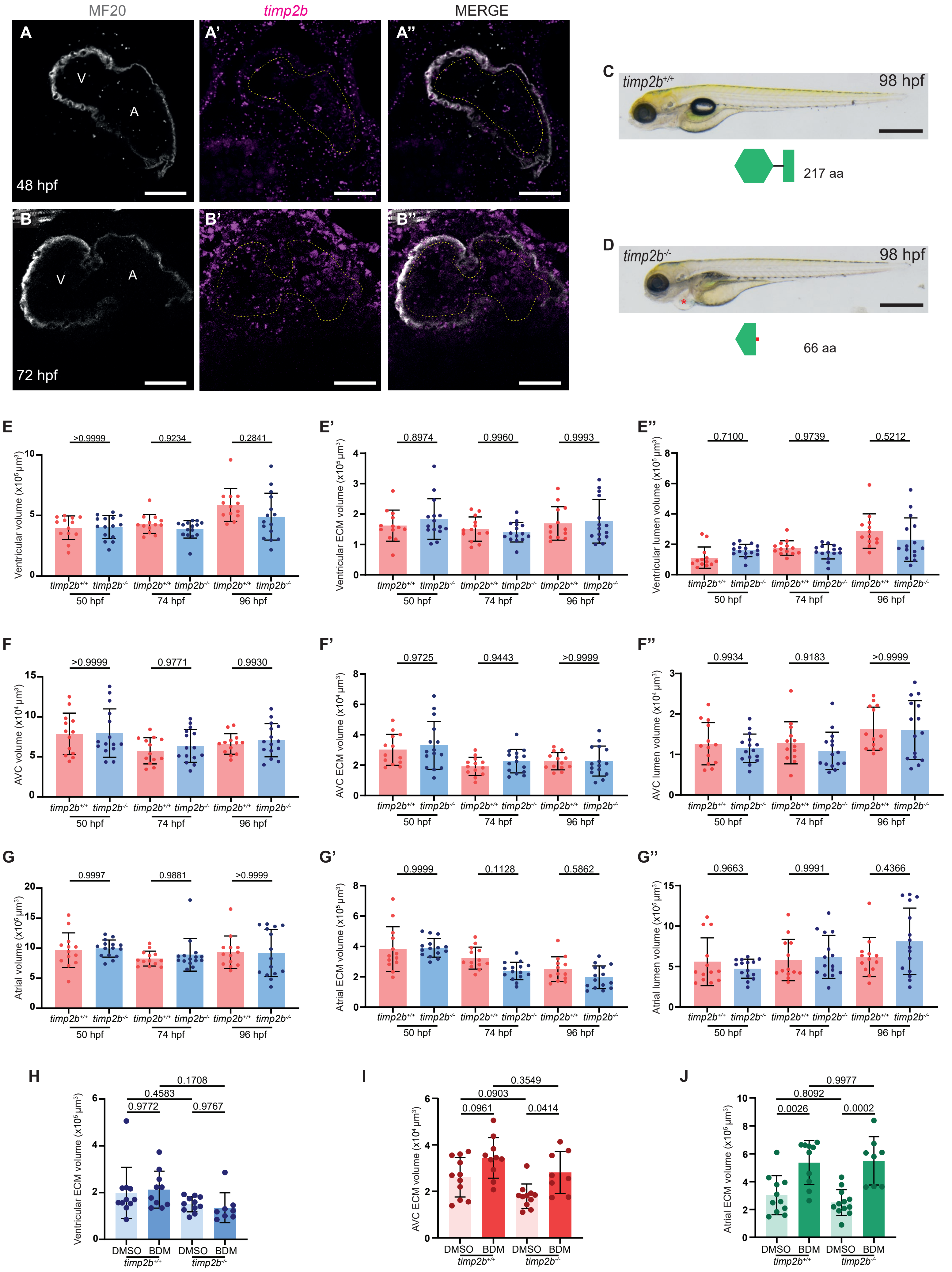

Development • Supplementary information

Fig. S3. Generation and characterization of *timp2b* loss-of-function mutants.
A-B'') Single-plane confocal images of *timp2b* mRNA expression as detected by fluorescence *in situ* hybridization in 48 (A-A'') and 72 (B-B'') hpf hearts. The endocardium is outlined by a yellow dashed line. *timp2b* expression was detected in endocardial cells and cardiomyocytes (MF20 staining in grey). **C-D)** Generation of *timp2b* loss-of-function mutation. Representative images of 98 hpf *timp2b*$^{+/+}$ and *timp2b*$^{-/-}$ larvae, and schematic of Timp2b proteins (C, D). In the wild-type Timp2b protein schematic (C), the hexagon and rectangle represent the N-terminal and C-terminal domains of Timp2, respectively, both of which are necessary for Timp binding to and inhibiting metalloproteinases. One guide RNA was used to generate a 5 base pair insertion in exon 2. The N-terminal domain of the Timp2b mutant protein is predicted to be truncated, resulting in a functionally null protein; red line in D indicates new sequence downstream of the premature termination codon. 98 hpf *timp2b*$^{-/-}$ larvae display pericardial edema (red asterisk) (D). **E-G'')** Quantification of ventricular (E-E''), AVC (F-F''), and atrial (G-G'') chamber, ECM and lumen volumes of *timp2b*$^{-/-}$ hearts compared with *timp2b*$^{+/+}$ sibling hearts. No significant differences were observed in the chamber, ECM, or lumen volumes. **H-J)** Quantification of total ventricular, AVC, and atrial ECM volumes in 74 hpf *timp2b*$^{+/+}$ and *timp2b*$^{-/-}$ hearts following 24 hour treatment with BDM compared with DMSO. Increased total cECM volume is observed in the AVC and atrium but not in the ventricle upon BDM treatment. n=13 for *timp2b*$^{+/+}$ and 15 for *timp2b*$^{-/-}$ (E-G''), n=11 for DMSO, *timp2b*$^{+/+}$, 10 for BDM, *timp2b*$^{+/+}$, 10 for DMSO, *timp2b*$^{-/-}$ and 8 for BDM, *timp2b*$^{-/-}$ (H-J); plot values represent means ± S.D.; p-values determined by one-way ANOVA followed by multiple comparisons with Dunn test. Scale bars: 50 µm (A-B''); 500 µm (C-D).

Development • Supplementary information

A

```
Timp2a      MPFSVNGILCTLALLVLWRPEELADACSCAPVHPQQAFCNADVVIRAKVVGEREVDSGND      60
Timp2b      MSM---SRSVPLLLLVLCGATDFAGACSCSPEHPQQAYCNADVVIRAKVVGRKEVVTGND      57
            * :     .  * ****    ::*.****:* *****:*************.:** :***

Timp2a      IYGNPIKRIQYEIKQIKMFKGPNQDIEAVFTAQVSAVCGVNLDATGKKEYLISGKAEADG      120
Timp2b      AYGYPIKMIRYDVKQLKMFKGPNGEIDTIFTGPSSALCGVNLESNGKQEYLITGNLNSNG      117
             ** *** *:*::**:******* :*:::**.  **:*****::.**:****:*: :::*

Timp2a      SMHVTLCDYIMPWDSLSATQKKSLSQRYQMGCECKIVRCPSLPCEISAPEECLWTDLMIE      180
Timp2b      TLRINLCDYIESWESLSLTQKKSLGPRYQMGCDCKIVQCPIIPCSISEPVECLWTDWVLE      177
            ::::.*****  *:*** ******. ******:****:** :**.** * ****** ::*

Timp2a      KQVHGRQANHYACVKRADGSCSWYRGVALPKKEFLDAEDP                          220
Timp2b      GTVQGTQAQHYTCVKRSDSSCAWYRGSTPPKKEFMDIEEP                          217
              *:* **:**:****:*.**:**** : *****:* *:*
```

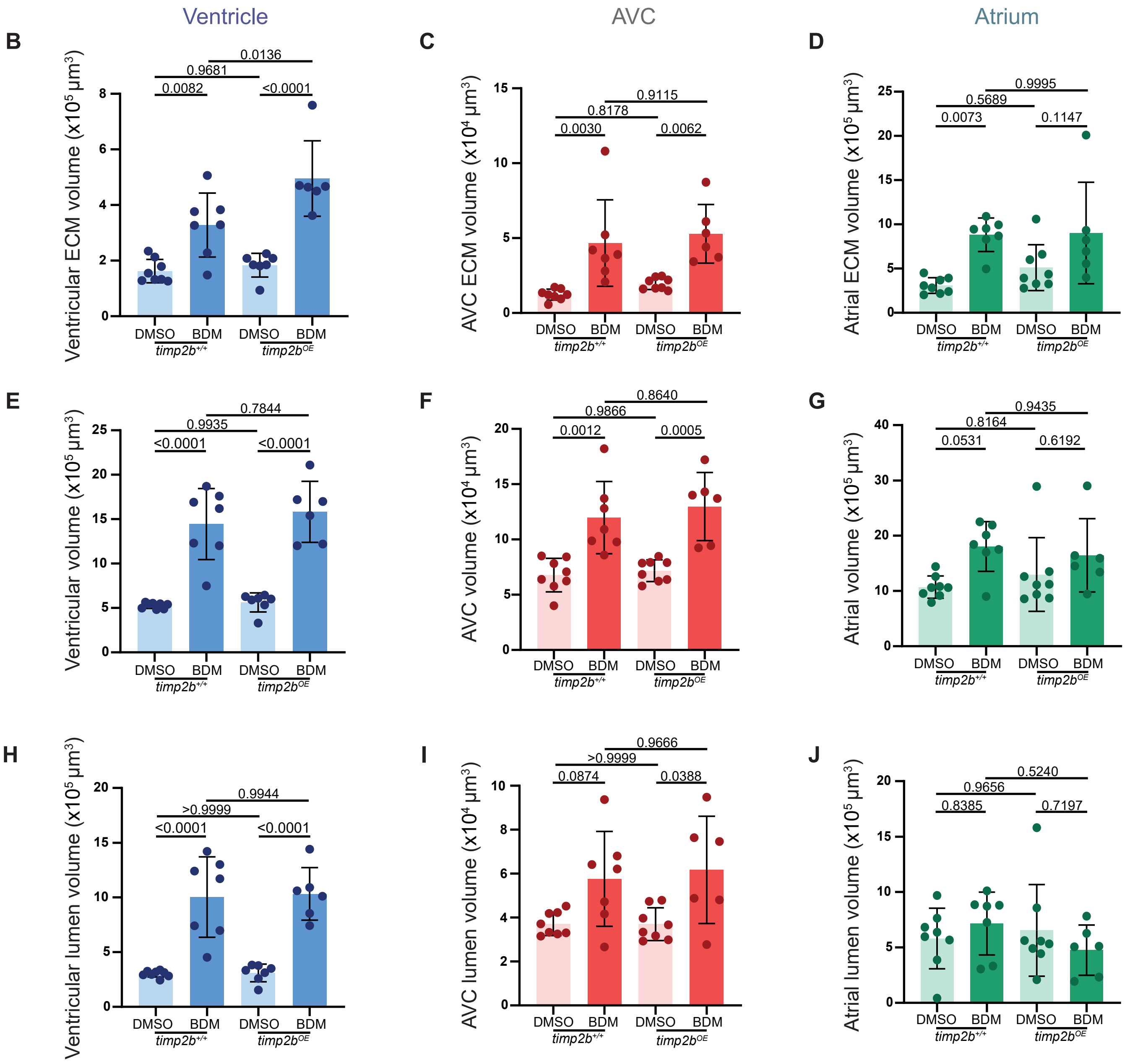

Fig. S4. Timp2a and Timp2b show high protein similarity.

A) Timp2a and Timp2b protein alignment shows 78% protein similarity. Red box indicates the Timp2 inhibitory domain; * indicates single, fully conserved residue; : indicates conservation between amino acid groups with similar properties; . indicates conservation between amino acid groups of weakly similar properties. Protein alignment was made using ClustalOmega (Madeira et al., 2022). **B-D)** Quantification of the total ventricular (B), AVC (C), and atrial (D) cECM volumes in 74 hpf control and *timp2b*-overexpressing hearts treated

Development • Supplementary information

with DMSO or BDM starting at 50 hpf. **E-G)** Quantification of the ventricular (E), AVC (F), and atrial (G) volumes, as marked by *Tg(myl7:*BFP-CAAX) expression in 74 hpf control and *timp2b*-overexpressing hearts treated for 24 hours with DMSO or BDM. **H-J)** Quantification of the ventricular (H), AVC (I) and atrial (J) lumen volumes of 74 hpf control and *timp2b*-overexpressing hearts treated for 24 hours with DMSO or BDM. n=8 for DMSO, $timp2b^{+/+}$, 7 for BDM, $timp2b^{+/+}$, 8 for DMSO, $timp2b^{OE}$ and 6 for BDM, $timp2b^{OE}$ (B-J); plot values represent means ± S.D.; Plot values represent means ± S.D.; p-values determined by one-way ANOVA followed by multiple comparisons with Dunn test.

Development • Supplementary information

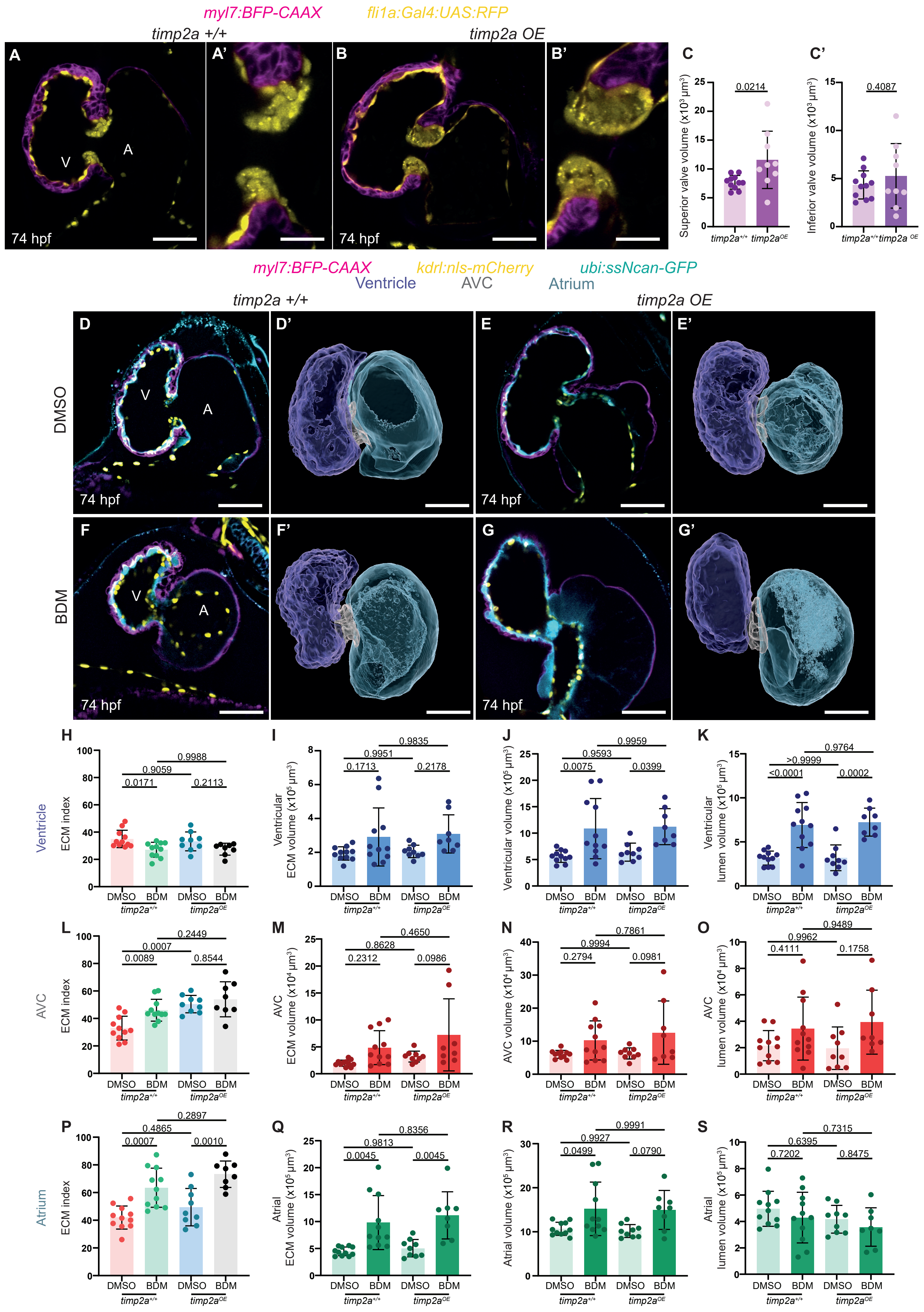

myl7:BFP-CAAX
fli1a:Gal4:UAS:RFP
timp2a +/+
timp2a OE
74 hpf
Superior valve volume (x10³ µm³)
Inferior valve volume (x10³ µm³)
0.0214
0.4087
myl7:BFP-CAAX
kdrl:nls-mCherry
ubi:ssNcan-GFP
Ventricle
AVC
Atrium
DMSO
BDM
ECM index
Ventricular ECM volume (x10⁵ µm³)
Ventricular volume (x10⁵ µm³)
Ventricular lumen volume (x10⁵ µm³)
AVC ECM volume (x10⁴ µm³)
AVC volume (x10⁴ µm³)
AVC lumen volume (x10⁴ µm³)
Atrial ECM volume (x10⁵ µm³)
Atrial volume (x10⁵ µm³)
Atrial lumen volume (x10⁵ µm³)

Fig. S5. Endocardial *timp2a* and *timp2b* overexpression exhibit similar phenotype.

A-B') Single-plane images and close-up of AV valves of 74 hpf control (A, A') and *timp2a* overexpressing (*Tg(fli1a:Gal4); Tg(UAS:timp2a-p2a-GFP*); B, B') hearts. Noticeable enlargement of the valve observed in *timp2a*-overexpressing hearts (B') compared with control (A'). **C', C')** Quantification of 3D valve tissue volumes in control and *timp2a* overexpressing larvae at 74 hpf. When *timp2a* is overexpressed in EdCs, significantly increased valve tissue volume is observed in the superior leaflet, but not in the inferior leaflet. **D-G')** Single-plane images and 3D surface rendering of 74 hpf control (D, D', F, F') and *timp2a* overexpressing (E, E', G, G') hearts treated with DMSO (D-E') or BDM (F-G') starting at 50 hpf. **H-K)** Quantification of ventricular ECM index (H), and total volumes of the ECM (I), chamber (J), and lumen (K) of 74 hpf control and *timp2a*-overexpressing hearts treated with DMSO or BDM starting at 50 hpf. **L-O)** Quantification of AVC cECM index (L), and total volumes of the ECM (M), chamber (N), and lumen (O) of 74 hpf control and *timp2a*-overexpressing hearts treated with DMSO or BDM starting at 50 hpf. Increased AVC cECM index (I) is observed in DMSO-treated *timp2a*-overexpressing hearts. **P-S)** Quantification of atrial cECM index (P), ECM (Q), chamber (R), and lumen volume (S) of 74 hpf control and *timp2a*-overexpressing hearts treated with DMSO or BDM starting at 50 hpf. n=11 for *timp2a*$^{+/+}$ and 9 for *timp2a*^{OE} (C, C'), n=11 for DMSO, *timp2a*$^{+/+}$, 11 for BDM, *timp2a*$^{+/+}$, 9 for DMSO, *timp2a*^{OE} and 8 for BDM, *timp2b*^{OE} (H-J); plot values represent means ± S.D.; p-values determined by unpaired two tailed Student's *t*-test (C,C') or one-way ANOVA followed by multiple comparisons with Dunn test (H-S). Scale bars: 20 µm (A, B), 10 µm (A', B'), 30 µm (D-G'). V, ventricle; A, atrium; AVC, atrioventricular canal.

Development • Supplementary information

Table S1. Summary of values of wild type treated with DMSO, BDM, or IBMX.

	Ventricular volume (x10^5 μm^3)	Ventricular lumen (x10^5 μm^3)	Ventricular ECM volume (x10^5 μm^3)	Ventricular ECM index (%)	AVC volume (x10^4 μm^3)	AVC lumen (x10^4 μm^3)	AVC ECM volume (x10^4 μm^3)	AVC ECM index (%)	Atrial volume (x10^5 μm^3)	Atrial lumen (x10^5 μm^3)	Atrial ECM volume (x10^5 μm^3)	Atrial ECM index (%)
**DMSO**	5.07	2.36	1.97	39.30	4.86	1.99	1.66	35.40	10.22	6.11	3.76	36.75
**BDM**	11.03	6.87	3.01	28.34	7.04	2.32	3.26	47.46	14.44	5.36	7.52	54.90
**IBMX**	7.04	3.88	1.89	27.30	5.66	1.91	1.86	32.92	13.62	7.26	4.54	33.93

Table S2. Summary of values of timp2b$^{-/-}$ treated with DMSO or BDM.

	Ventricular volume (x10^5 μm^3)	Ventricular lumen (x10^5 μm^3)	Ventricular ECM volume (x10^5 μm^3)	Ventricular ECM index (%)	AVC volume (x10^4 μm^3)	AVC lumen (x10^4 μm^3)	AVC ECM volume (x10^4 μm^3)	AVC ECM index (%)	Atrial volume (x10^5 μm^3)	Atrial lumen (x10^5 μm^3)	Atrial ECM volume (x10^5 μm^3)	Atrial ECM index (%)
**DMSO**	5.45	2.09	1.98	35.86	7.21	1.66	2.61	35.99	9.14	4.92	3.02	29.27
**BDM**	5.86	2.53	2.12	36.04	7.72	1.31	3.44	46.03	8.32	5.70	5.36	54.52

Table S3. Summary of values of *timp2b*^{OE} treated with BDM compared with DMSO control.

	Ventricular volume (x10^5 μm^3)	Ventricular lumen (x10^5 μm^3)	Ventricular ECM volume (x10^5 μm^3)	Ventricular ECM index (%)	AVC volume (x10^4 μm^3)	AVC lumen (x10^4 μm^3)	AVC ECM volume (x10^4 μm^3)	AVC ECM index (%)	Atrial volume (x10^5 μm^3)	Atrial lumen (x10^5 μm^3)	Atrial ECM volume (x10^5 μm^3)	Atrial ECM index (%)
**DMSO**	6.40	3.19	2.05	33.20	6.30	1.96	3.18	50.46	10.18	4.16	5.06	49.47
**BDM**	11.23	7.24	3.09	27.49	12.59	3.93	7.22	53.96	14.96	3.57	11.15	73.25

Table S4. Summary of values of *timp2a*^{OE} treated with BDM compared with DMSO control.

	Ventricular volume (x10^5 μm^3)	Ventricular lumen (x10^5 μm^3)	Ventricular ECM volume (x10^5 μm^3)	Ventricular ECM index (%)	AVC volume (x10^4 μm^3)	AVC lumen (x10^4 μm^3)	AVC ECM volume (x10^4 μm^3)	AVC ECM index (%)	Atrial volume (x10^5 μm^3)	Atrial lumen (x10^5 μm^3)	Atrial ECM volume (x10^5 μm^3)	Atrial ECM index (%)
**DMSO**	5.62	3.02	1.83	32.45	7.17	3.70	1.95	27.36	12.99	6.54	5.10	39.30
**BDM**	15.81	10.32	4.95	31.82	12.98	6.17	5.28	40.12	16.44	4.75	9.02	51.62

Development • Supplementary information

Table S5. Primer list

	Gene name	Forward	Reverse	
PCR	*timp2a*	ATGAAGAGCGTCAGGAGCTGT	AGGGTCTTCCACATCCA	To generate the overexpression plasmid
PCR	*timp2b*	GTCGACCACCATGAGTATGTC	AGGGTCTTCCACATCCA	To generate the overexpression plasmid
qPCR	*timp2b*	CGTGAAGATAGTGTCAATCTCTC	TCACCGGCAATGACGCTTATG	
qPCR	*timp2a*	ATAAGCATGCGCTGAGGAAGAGGA	AGCTGCAACAATCCAACTCCATGC	
qPCR	*mmp2*	AGCTTTGACGATGACCGCAAATGG	TCAGAATGCTCTAAACCCAGGGCA	
qPCR	*mmp14a*	GTGTTTCTGGTGCAGAGCG	CCGAGATAGCGGAGTTGATAG	
qPCR	*mmp14b*	CTGGAGCGGGTTTACGAGG	CATGGCAGCAATGGCAGAG	
qPCR	*rpl13a*	TCTGGAGGACTGTAAGAGGTATGC	AGACGCACAATCTTGAGAGCAG	
HRM	*timp2b*	AGCAAAAGTCGTCGGAAGAA	TTTGACATCGTATCGGATCA	To genotype *timp2b* mutants

Table S6. Ct values of genes by RT-qPCR

	50 hpf		74 hpf		98 hpf		74 hpf	
**Gene**	Uninjected	*tnnt2a* MO	Uninjected	*tnnt2a* MO	Uninjected	*tnnt2a* MO	DMSO	BDM
timp2b	30.13	29.71	29.84	28.85	31.15	25.66	27.11	22.40
timp2a	27.61	27.62	26.89	27.20	27.51	27.27	25.40	25.05
mmp2	27.93	28.53	27.62	27.29	29.10	29.03	24.52	24.50
mmp14a	28.56	28.81	28.17	28.18	29.62	29.74	25.37	25.59
mmp14b	24.06	23.94	23.66	23.87	26.79	24.32	23.25	22.82
rpl13a	19.44	19.46	19.24	19.15	20.63	19.35	18.87	18.56
	50 hpf		74 hpf		98 hpf		74 hpf	
**Gene**	DMSO	IBMX	DMSO	IBMX	DMSO	IBMX	DMSO	0.1% Tricaine
timp2b	27.25	26.57	27.68	27.91	28.58	28.29	29.45	27.79
rpl13a	19.88	19.13	20.60	21.08	21.71	21.57	22.16	22.42

Development • Supplementary information
